# Overexpressed KCNK1 regulates potassium channels affecting molecular mechanisms and biological pathways in bladder cancer

**DOI:** 10.1186/s40001-024-01844-1

**Published:** 2024-04-30

**Authors:** Wei Zhang, Xiao-Song Chen, Ying Wei, Xiao-Min Wang, Xian-Jin Chen, Bang-Teng Chi, Lin-Qing Huang, Rong-Quan He, Zhi-Guang Huang, Qi Li, Gang Chen, Juan He, Mei Wu

**Affiliations:** 1 Department of Pathology, The First Affiliated Hospital of Guangxi Medical University, 6 Shuangyong RD, Nanning, 530021 Guangxi Zhuang Autonomous Region People’s Republic of China; 2 Department of Medical Oncology, The First Affiliated Hospital of Guangxi Medical University, 6 Shuangyong RD, Nanning, 530021 Guangxi Zhuang Autonomous Region People’s Republic of China

**Keywords:** Gene expression, KCNK1, Biological functions, Molecular mechanism, Potassium channel

## Abstract

**Background:**

This study aimed to explore the expression, molecular mechanism and its biological function of potassium two pore domain channel subfamily K member 1 (KCNK1) in bladder cancer (BC).

**Methods:**

We integrated large numbers of external samples (n = 1486) to assess KCNK1 mRNA expression levels and collected in-house samples (n = 245) for immunohistochemistry (IHC) experiments to validate at the KCNK1 protein level. Single-cell RNA sequencing (scRNA-seq) analysis was performed to further assess KCNK1 expression and cellular communication. The transcriptional regulatory mechanisms of KCNK1 expression were explored by ChIP-seq, ATAC-seq and ChIA-PET data. Highly expressed co-expressed genes (HECEGs) of KCNK1 were used to explore potential signalling pathways. Furthermore, the immunoassay, clinical significance and molecular docking of KCNK1 were calculated.

**Results:**

KCNK1 mRNA was significantly overexpressed in BC (SMD = 0.58, 95% CI [0.05; 1.11]), validated at the protein level (p < 0.0001). Upregulated KCNK1 mRNA exhibited highly distinguishing ability between BC and control samples (AUC = 0.82 [0.78–0.85]). Further, scRNA-seq analysis revealed that KCNK1 expression was predominantly clustered in BC epithelial cells and tended to increase with cellular differentiation. BC epithelial cells were involved in cellular communication mainly through the MK signalling pathway. Secondly, the KCNK1 transcription start site (TSS) showed promoter-enhancer interactions in three-dimensional space, while being transcriptionally regulated by GRHL2 and FOXA1. Most of the KCNK1 HECEGs were enriched in cell cycle–related signalling pathways. KCNK1 was mainly involved in cellular metabolism–related pathways and regulated cell membrane potassium channel activity. KCNK1 expression was associated with the level of infiltration of various immune cells. Immunotherapy and chemotherapy (docetaxel, paclitaxel and vinblastine) were more effective in BC patients in the high KCNK1 expression group. KCNK1 expression correlated with age, pathology grade and pathologic_M in BC patients.

**Conclusions:**

KCNK1 was significantly overexpressed in BC. A complex and sophisticated three-dimensional spatial transcriptional regulatory network existed in the KCNK1 TSS and promoted the upregulated of KCNK1 expression. The high expression of KCNK1 might be involved in the cell cycle, cellular metabolism, and tumour microenvironment through the regulation of potassium channels, and ultimately contributed to the deterioration of BC.

**Supplementary Information:**

The online version contains supplementary material available at 10.1186/s40001-024-01844-1.

## Introduction

Bladder cancer (BC) is globally one of the most serious urological malignancies and has been classified as non-muscle-invasive BC and muscle-invasive BC [1, 2]. BC may be induced by both smoking and long-term exposure to paint-rich components (polycyclic aromatic hydrocarbons, benzene, aromatic amines, etc.) [3]. In recent years, AI-based bladder cancer screening and diagnosis has been widely studied [4]. As the fifth most common cancer in the United States, 83,190 new cases of BC and up to 16,840 deaths are expected in 2024 [5]. Currently, cystectomy combined with neoadjuvant therapy (chemotherapy and immunotherapy) remains the first-line treatment for BC [6]. Cisplatin-based chemotherapy has shown initial anti-tumour activity [7]. In addition, immune checkpoint inhibitors, BCG, and platelet-rich plasma are often used for immunotherapy [8–10]. Although neoadjuvant therapy has brought new options for BC treatment, side effects such as inflammation and organ function abnormalities still occur clinically [11]. Therefore, further exploration of the developmental mechanisms and therapeutic targets associated with BC is essential for the clinical management of BC patients.

Potassium two pore domain channel subfamily K member 1 (KCNK1) is a widely expressed pH-gated two pore structural domain potassium channel, often encoding TWIK-1 or K2P1 protein[12, 13]. KCNK1 plays an important role in physiological and pathological functions associated with changes in electrical membrane potential by controlling the efflux of potassium ions to maintain the balance of the resting potential of the cell membrane [14, 15]. Previous research indicated that KCNK1 plays a very vital role in promoting tumour cell malignant transformation, especially in regulating cell cycle progression and cancer cell malignant proliferation and migration [16]. In addition, KCNK1 is highly expressed in thyroid cancer [16], breast cancer (BRCA) [17], non-functioning pituitary adenoma [18], and pancreatic ductal adenocarcinoma [19], and it can be used as a diagnostic and relevant prognostic marker for tumours. In thyroid cancer, KCNK1 can promote tumour malignancy through cell cycle, PI3K and MAPK signalling pathways [16]. Promoter hypermethylation causes aberrant expression of KCNK1 and promotes BRCA cell proliferation and migration [20]. Nevertheless, studies on the differential expression of KCNK1 in BC are still lacking, and the molecular mechanisms and biological functions of KCNK1 in BC are unclear. Therefore, it is necessary to investigate the role of KCNK1 in BC at a deeper level.

In the present study, overexpression of KCNK1 was confirmed by global BC transcriptome data and immunohistochemistry (IHC) experiments. Single-cell RNA sequencing (scRNA-seq) was used to further analyse KCNK1 expression as well as cellular communication. Next, we discussed the molecular mechanisms and biological functions of KCNK1 expression in BC. Finally, we assessed the correlation of KCNK1 expression with the tumour microenvironment (TME) and clinical significance. These results contribute to an in-depth understanding of KCNK1 expression and its molecular mechanisms, and have important implications for the prevention and treatment of BC patients.

## Materials and methods

### Screening and processing of transcriptional profiles

In the present study, the datasets that were included were from ArrayExpress, Sequence Read Archive, The Cancer Genome Atlas (TCGA), and Gene Expression Omnibus (GEO). The inclusion exclusion steps for BC–related microarray and RNA-seq data were shown in Additional file 2: Figure S1. There were 21 datasets finally included in this study (Table 1). The 21 included datasets were log2(x + 1) processed and normalised by the ‘limma’ package [21], and we merged the datasets with the same experimental platform. In the end, we obtained 13 completely new cohorts, and the ‘SVA’ package [22] was used to eliminate batch effects.

**Table 1:** Basic information about the high-throughput sequencing datasets included in this study

Category	Dataset	Platform	Country	Year	BC samples	Non-BC samples
tissue mRNA	E-MTAB-1940	–	France	2015	82	4
	GSE65635	GPL14951	Russia	2015	8	4
	GSE86411	GPL14951	USA	2016	132	0
	GSE7476	GPL570	Spain	2007	9	3
	GSE31684	GPL570	USA	2012	93	0
	GSE2109	GPL570	USA	2005	15	8
	GSE31189	GPL570	USA	2013	52	40
	GSE19423	GPL6102	South Korea	2010	48	0
	GSE37815	GPL6102	South Korea	2013	18	6
	GSE13507	GPL6102	South Korea	2010	165	68
	GSE2361	GPL96	USA	2005	0	1
	GSE3167	GPL96	Denmark	2005	41	9
	GSE5287	GPL96	Denmark	2007	30	0
	GSE19915 _ GPL3883	GPL3883	Sweden	2010	76	8
	GSE236932	GPL24676	China	2023	38	25
	GSE24152	GPL6791	USA	2010	10	7
	GSE40355	GPL8227	Germany	2013	16	8
	GSE51843	GPL10558	Spain	2014	5	6
	GSE52519	GPL6884	Russia	2013	9	3
	GSE76211	GPL17586	China	2017	3	3
	TCGA_BLCA_mRNA	–	–	–	414	19

*BC* bladder cancer

### IHC staining of in-house samples

We collected 245 in-house tissue samples (199 BC and 46 control samples) from the First Affiliated Hospital of Guangxi Medical University and made them into four tissue microarrays (No. BLC1501, BLC481, BLC1021, and BLC242, Guilin Pansum Bio-technology Company, Guangxi Zhuang Autonomous Region, China). Then, IHC staining was performed in strict accordance with the standard. First, the tissue sections were formalin-fixed and paraffin-embedded. The sections were deparaffinised and antigenically repaired using ethylenediaminetetraacetic acid buffer. Next, we inactivated endogenous peroxidase activity and treated with KCNK1 rabbit polyclonal antibody (Abcam; ab224381; dilution ratio 1:100) (stored in 4 ℃). The tissue microarrays were finally re-incubated, stained, dehydrated and sealed at room temperature. Phosphate-buffered saline was used for washing during the experiments. The staining results of the sections were defined into 4 scores: 0 (no staining); 1 (pale yellow); 2 (brownish yellow); and 3 (brownish black). The percentage of positive cells in the sections was defined into 5 scores: 0 (number at ≤ 5%); 1 (number at 6–25%); 2 (number at 26–50%); 3 (number at 51–75%); and 4 (number at ≥ 76%). The total IHC score was assessed separately by two senior pathologists (the product of the percentage of positive cells and the staining score), and the raw data of the experiments were provided in Additional file 1. All patients signed an informed consent form and the Ethics Committee of the First Affiliated Hospital of Guangxi Medical University approved our study (2023-S058-01).

### scRNA-seq analysis of KCNK1 in BC tissues

The dataset GSE190888 for BC-associated scRNA-seq analysis is available from GEO. The ‘Seurat’ package was used for pre-processing and screening of the data, filtering out genes expressed in less than 3 cells and cells expressing less than 50 genes. Immediately after that, we performed quality control on the filtered data, retaining cells with gene expression greater than 500 and mitochondrial content less than 25%. After data normalisation, PCA downscaling analysis was performed. We selected the top 20 PCs for cluster analysis, and the uniform manifold approximation and projection (UMAP) algorithm was used for secondary dimensionality reduction, and the ‘SingleR’ package [23] was used for cell type annotation. Further, the ‘CytoTRACE’ [24] and ‘CellChat’ [25] packages were used for cellular temporal analysis and cellular communication analysis, respectively. In addition, the ‘AUCell’ package allowed the calculation of AUC values for each cell and the assessment of single-cell metabolic activity.

### Potential epigenetic regulatory mechanisms of KCNK1 expression

To further explore the potential regulatory mechanisms of KCNK1 expression, we predicted the transcription factor (TF) of the upstream transcription start site (TSS) of KCNK1. Chromatin immunoprecipitation sequencing (ChIP-seq) of TF in Cistrome Data Browser was used to validate the above predictions. Assay for Transposase-Accessible Chromatin sequencing (ATAC-seq) and ChIP-seq of multiple histone modifications were derived from Cistrome Data Browser. Chromloops is a novel database that aggregates a large number of PLAC-seq, ChIA-PET, and HiChIP datasets, which are often used in studies of chromatin interaction regulation [26]. In this study, the ChIA-PET dataset of Homo sapiens HT-1197 was included and RAD21 was identified as a ChIP-seq marker.

### Potential signalling pathways of KCNK1 in BC

Co-expressed genes of KCNK1 in BC were identified by Pearson correlation coefficient *r*. If the Pearson correlation coefficient *r* ≥ 0.3, *p* < 0.05 and a gene met this condition in at least three datasets, this indicated that the gene may be co-expressed with KCNK1. The combined standardised mean difference (SMD) of the included BC datasets was calculated, and SMD > 0, *p* < 0.05 for highly expressed genes in BC. Highly expressed genes and KCNK1 co-expressed genes in BC tissues were intersected, and the intersection results were defined as highly expressed co-expression genes (HECEGs) of KCNK1. To further explore the potential molecular mechanisms of KCNK1 in BC, we performed pathway enrichment analysis with HECEGs. The ‘ReactomePA’ package [27] was used for Reactome enrichment analysis, and the ‘clusterProfiler’ package [28] was used for KEGG and GO enrichment analysis.

In addition, this study downloaded a subset of KEGG files from the Molecular Signatures Database. Based on the dataset TCGA_BLCA_mRNA, we evaluated the biological pathways and molecular mechanisms associated with KCNK1 expression. The protein–protein interaction network (PPI) of KCNK1 could be constructed by GeneMANIA (https://genemania.org/).

### Immunocorrelation analysis of KCNK1 expression

The ‘IOBR’ package [29] is a multi-omics immuno-oncology biology R package for exploring TME. The CIBERSORT algorithm was used to demonstrate the composition of TME in BC and the correlation of KCNK1 expression with 22 immune cell scores. The TIMER algorithm was commonly used to analyse the correlation of KCNK1 expression with the levels of 6 common immune cells. Further, we used the ESTIMATE algorithm to comprehensively explore BC TME for immune cell, stromal cell and tumour purity.

### Clinically relevant analyses and molecular docking

To elucidate the clinical significance of KCNK1 expression, we performed a comprehensive analysis of treatment outcomes and clinicopathological characteristics of BC patients. In this study, based on The Cancer Immunome Database (https://tcia.at/home), we predicted the effect of anti-CTLA-4 and anti-PD-1 in BC patients by immunophenotypic scores. The TIDE score was calculated from the Tumor Immune Dysfunction and Exclusion (http://tide.dfci.harvard.edu/) database, reflecting the likelihood of immune escape in BC patients. Meanwhile, the ‘oncoPredict’ package [30] was used for drug sensitivity analysis, and the related files could be downloaded from Genomics of Drug Sensitivity in Cancer v2.0.

In addition, we downloaded the crystal structure of KCNK1 (PDB ID: 3UKM) and the 2D structure of the drug molecule from the RCSB PDB database and PubChem database, respectively, followed by free energy minimisation done by ChemBio3D Ultra. The KCNK1 protein active site was predicted using POCASA 1.1 (https://g6altair.sci.hokudai.ac.jp/g6/service/pocasa/). Meanwhile, the drug molecules and KCNK1 protein structures were processed using AutoDockTools software, and molecular docking was performed using QuickVina-w software under Linux environment. The results of molecular docking were presented in affinity and visualisation was done by Discovery Studio and PyMOL.

### Statistical analysis

**Fig. 1:**
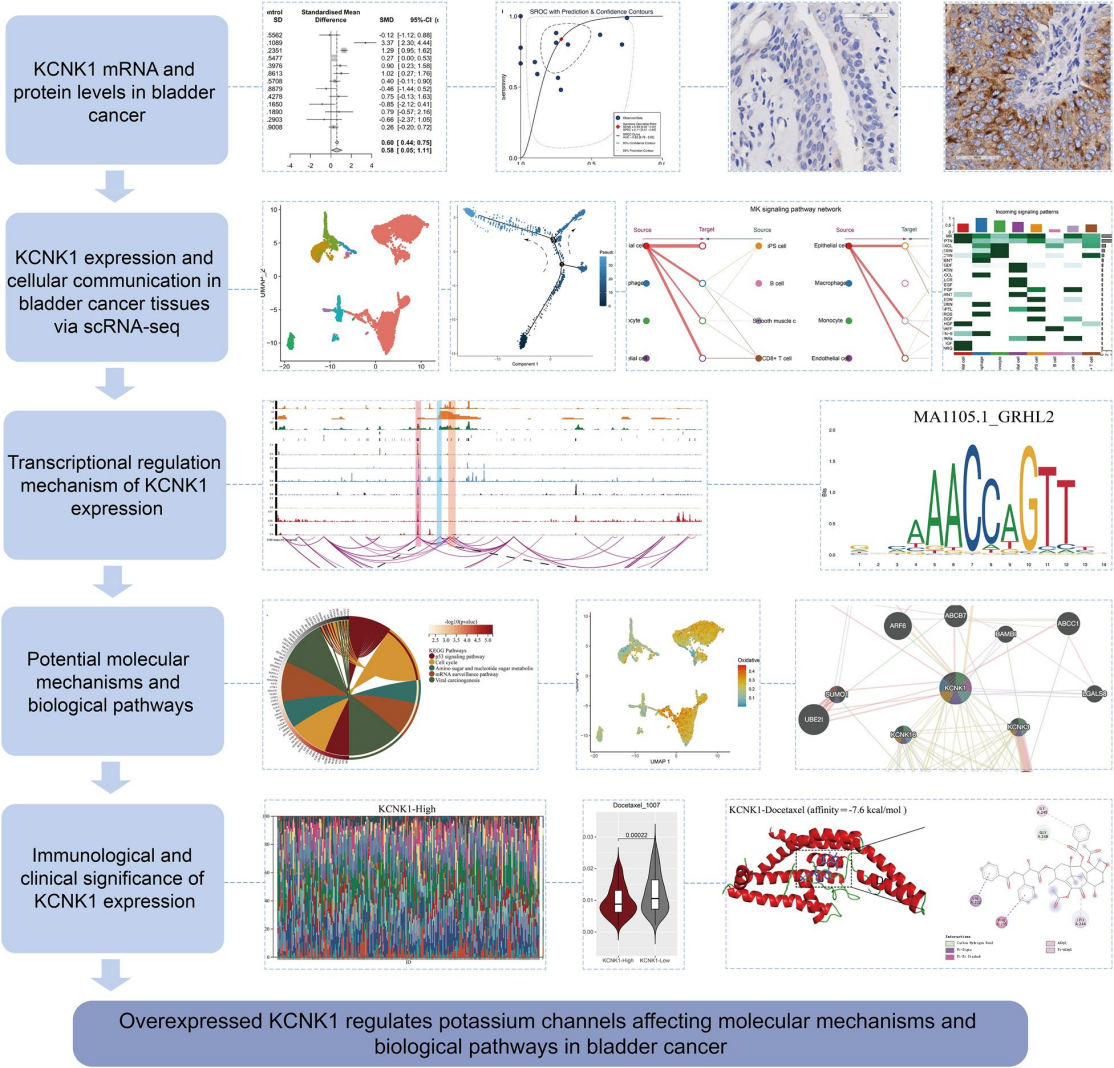
The research overflow of this study

In this study, *p* < 0.05 indicated that the results were statistically significant. If the heterogeneity between datasets is too large (*I*^*2*^ > 50% and *p* ≤ 0.05), a random effects model should be chosen to assess SMD. SMD results could be considered statistically significant when the 95% confidence interval (CI) did not contain zero. Differences within groups were compared by The Wilcoxon rank-sum test. The *p* > 0.1 in Egger’s test indicated that there was no significant publication bias in the SMD results. The t-test and ANOVA analyses were used to compare means of two or more samples in clinicopathological characteristics, respectively. The area under the curve (AUC) of the receiver operator characteristic (ROC) curve and the summary receiver operator characteristic (sROC) curve were used to assess the efficacy of the target to discriminate between cancer and control samples. The Stata (v18.0), GraphPad Prism 10.0, SPSS 23.0, and R (v4.3.1) were used for the above calculation and visualisation steps. The steps of the present study were displayed in Fig. 1.

## Results

### KCNK1 mRNA expression was significantly upregulated in BC tissues

**Fig. 2:**
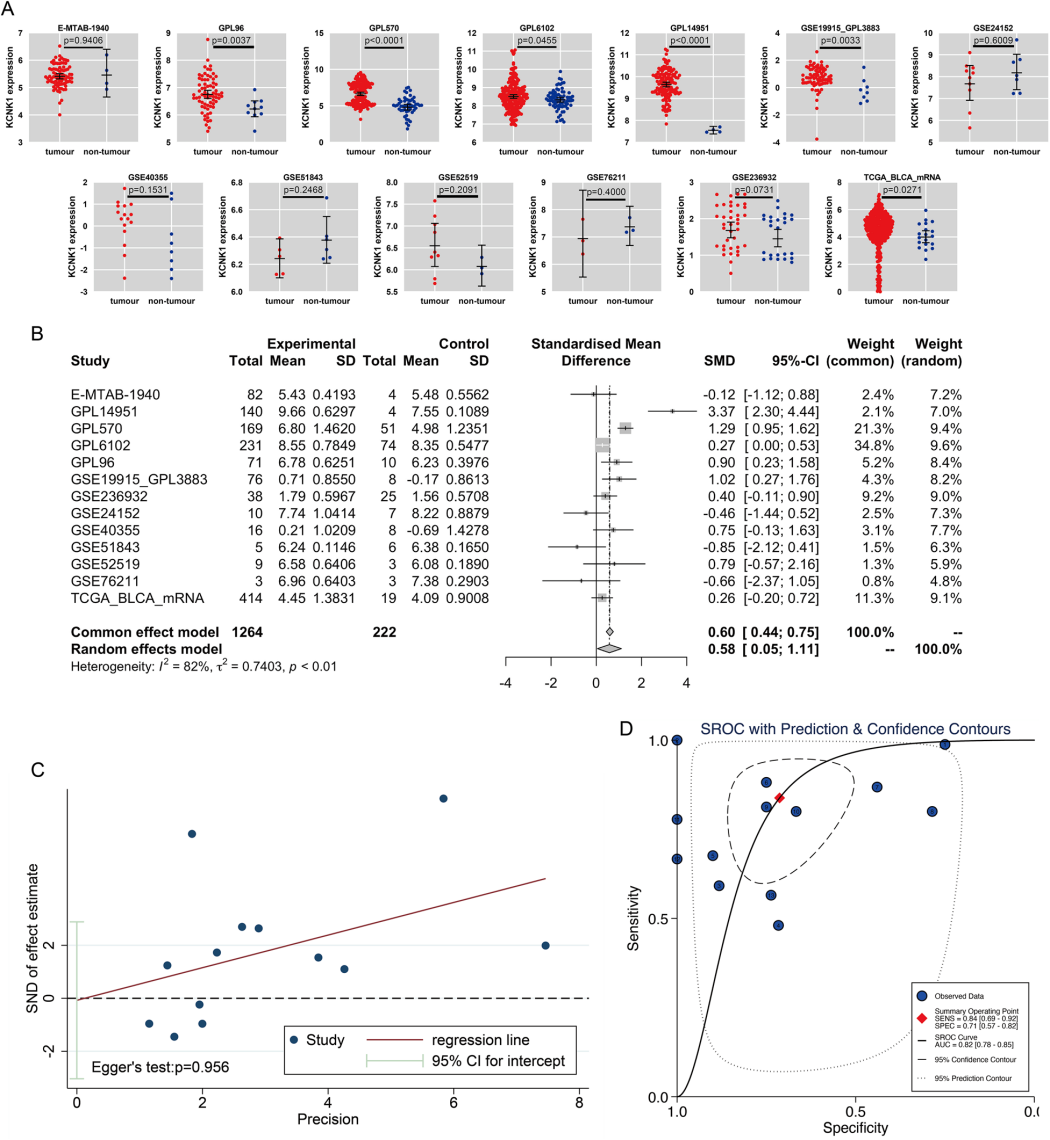
Comparison of differences in KCNK1 mRNA expression levels in control and bladder cancer groups. **A** KCNK1 mRNA was differentially expressed in each dataset. **B** Forest plot of standardised mean difference of KCNK1 mRNA in bladder cancer tissues. **C** Egger’s test. **D** Summary receiver operator characteristic

Among the 13 datasets merged in this study, six datasets (GPL96, GPL570, GPL6102, GPL14951, GSE19915_GPL3883, and TCGA_BLCA_mRNA) demonstrated that KCNK1 mRNA was significantly elevated in BC (*p* < 0.05; Fig. 2A). Pooling the datasets to calculate the composite SMD, KCNK1 mRNA was significantly increased in 1264 BC samples compared to 222 control samples (SMD = 0.58, 95% CI [0.05; 1.11]; Fig. 2B). In Fig. 2C, Egger’s test indicated that there was no publication bias in the results of SMD (*p* = 0.956). Considering that KCNK1 mRNA expression was significantly different in cancer and control samples, we plotted the ROC of each dataset to assess the distinguishing ability of KCNK1 mRNA. The results showed that KCNK1 mRNA expression had an extremely strong ability in distinguishing cancer and control samples (Additional file 2: Figure S2A), which was further validated by the results of the combined sROC (AUC = 0.82 [0.78–0.85]; Fig. 2D).

### KCNK1 protein levels in BC were detected by in-house IHC

**Fig. 3:**
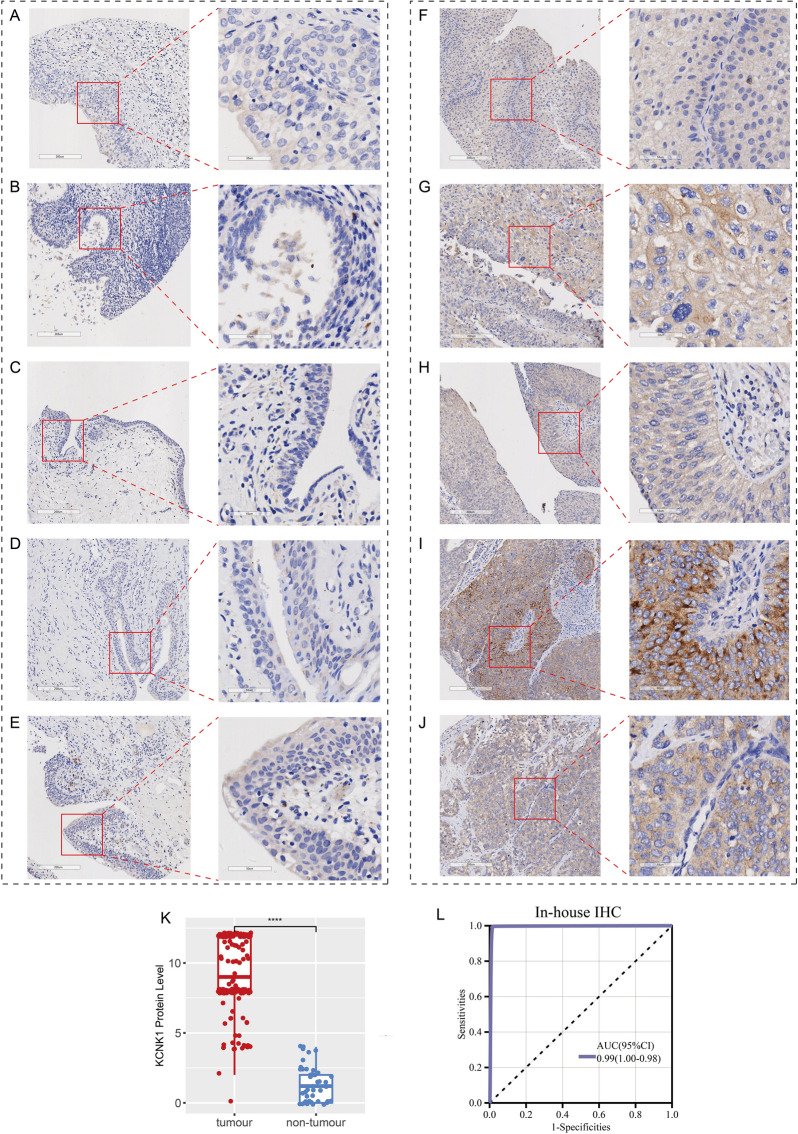
KCNK1 protein levels were assessed by immunohistochemistry (IHC) assay based on in-house bladder cancer samples. **A**–**E** IHC staining of control samples. **F**–**J** IHC staining of cancer samples. **K** Protein levels of KCNK1 in bladder cancer and control samples. **L** Receiver operator characteristic curves of in-house IHC. ^ns/NS^p > 0.05, ^∗^p < 0.05, ^∗∗^p < 0.01, ^∗∗∗^p < 0.001, ^∗∗∗∗^p < 0.0001

Further, we performed IHC staining of in-house samples (n = 245) to verify KCNK1 expression in BC tissues at the protein level. According to Fig. 3A–E, KCNK1 protein stained weakly positive in control tissue samples under the microscope. Compared with the control samples, KCNK1 protein stained strongly positive in BC tissue samples by IHC (Fig. 3F–J), and the protein level was significantly elevated (*p* < 0.0001; Fig. 3K). The ROC curves indicated that at the protein level, highly expressed KCNK1 also possessed an excellent ability to distinguish between BC and control samples (AUC = 0.99 [1.00–0.98]; Fig. 3L).

### scRNA-seq revealed KCNK1 expression and cellular communication

**Fig. 4:**
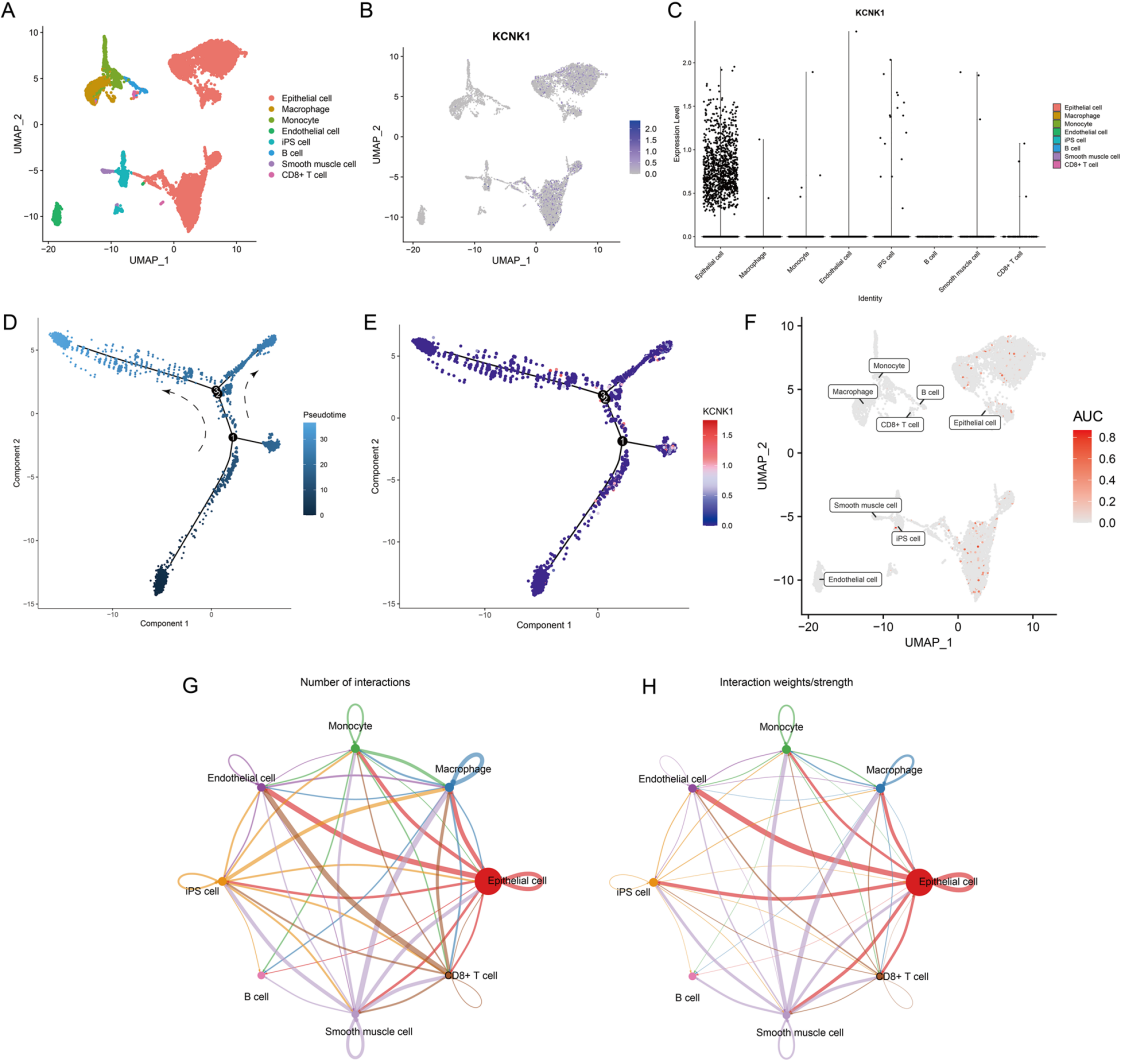
Comprehensive analysis of single-cell RNA sequencing exploring KCNK1 expression. **A** Included cells were annotated into 8 cell types. **B** UMAP plot of KCNK1 expression in cells. **C** Violin plots of KCNK1 expression in different cells. **D** The cellular differentiation trajectory in pseudo-temporal analysis. **E** Expression levels of KCNK1 at cell differentiation. **F** Mapping of AUC values of each cell onto UMAP plots. **G** Number of cell interactions. **H** Cell interaction weights/strengths

Immediately following this, we further analysed the distribution of KCNK1 expression in cells of BC tissues and intercellular communication using scRNA-seq. This study filtered to obtain 9255 cells annotated as 8 common cell types (Fig. 4A). KCNK1 was abundantly expressed in BC epithelial cells (Fig. 4B, C), and there was a trend of elevated KCNK1 expression with cell differentiation (Fig. 4D, E). AUCell analysis showed that KCNK1 was mainly active in epithelial cells (Fig. 4F).

**Fig. 5:**
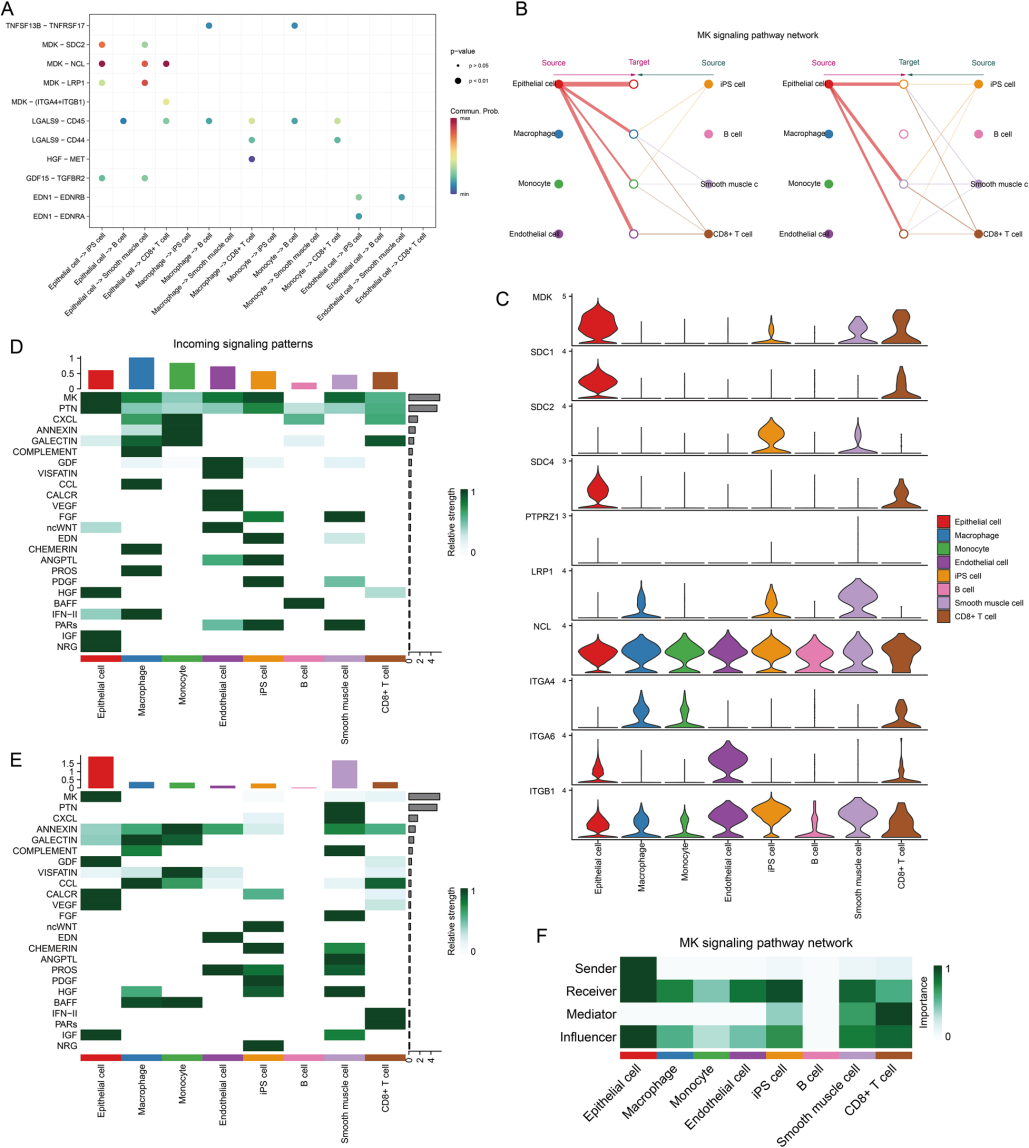
Cell communication analysis based on the ‘Cellchat’ package. **A** Ligand-receptor pair interactions in cellular communication. **B** Hierarchical structure of cell clusters involved in the MK signalling pathway. **C** Distribution of signalling genes involved in the MK signalling pathway. **D** Afferent signalling contributions of different cell clusters. **E** Different cell cluster efferent signalling contributions. **F** Signalling roles of different cell clusters in the MK signalling pathway

Considering the large number and weight/intensity of different types of cellular interactions in BC tissues (Fig. 4G, H), we further analysed the specific mechanisms of cellular communication. First, among all ligand-receptor (L-R) pair interactions, the MDK-associated L-R pair was very active in BC epithelial cells (Fig. 5A). Since MDK was active in the MK signalling pathway, we launched a specific analysis of the MK signalling pathway. Figure 5B, C showed that the MK signalling pathway was mainly involved in cellular communication between epithelial cells and iPS cells, Smooth muscle cells and CD8 + T cells, and genes involved in the MK signalling pathway were also mainly expressed in the above cells. MK signalling pathway contributed the greatest intensity among all afferent and efferent signals (Fig. 5D, E). Among them, BC epithelial cells were the main sender and receiver, and CD8 + T cells were the main mediator (Fig. 5F). In addition, BC epithelial cells were identified from the global communication pattern to be mainly involved in Pattern 2. The major signalling to contributors included MK, ANNEXIN, GALECTIN, CCL, BAFF and IFN-II (Additional file 2: Figure S3).

### Potential epigenetic regulatory mechanisms of KCNK1 overexpression

**Fig. 6:**
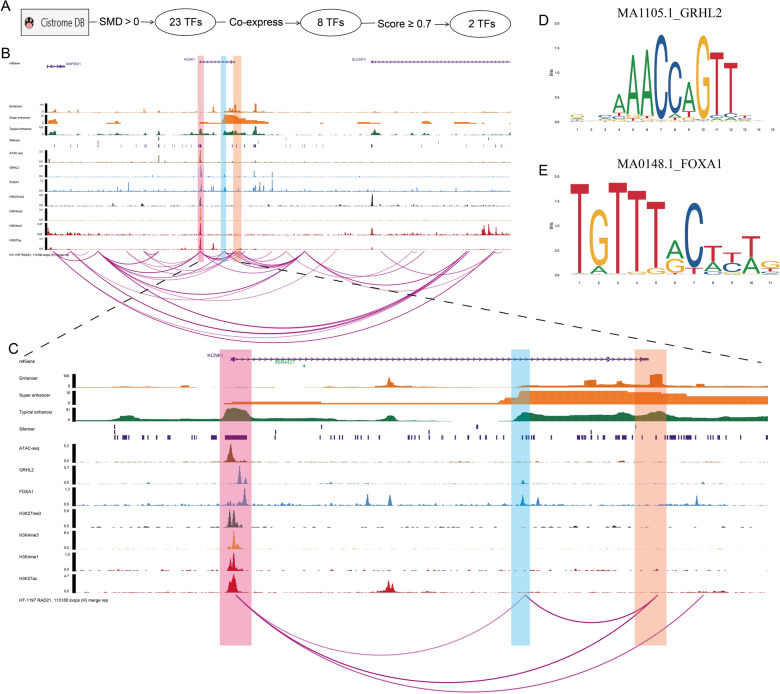
Epigenetic regulatory mechanisms of KCNK1 expression. **A** Cistrome Data Browser-based transcription factor screening process. **B** Multi-omics exploration of potential regulatory mechanisms of KCNK1 (before amplification). **C** Multi-omics exploration of potential regulatory mechanisms of KCNK1 (after zoom-in). **D** Motif map of transcription factor GRHL2. **E** Motif map of the transcription factor FOXA1

In the present study, we explored the potential regulatory mechanism of KCNK1 in depth to reveal the cause of elevated KCNK1 expression. We used Cistrome Data Browser to predict KCNK1 upstream TFs (Fig. 6A) and obtained two TFs (GRHL2 and FOXA1) after a series of screening. The ChIP-seq of these two TFs possessed peaks at 2 kb before and after the KCNK1 upstream TSS, suggesting that GRHL2 and FOXA1 might act on the KCNK1 TSS and regulate the upregulation of KCNK1 expression (Fig. 6B, C). The motif plots of GRHL2 and FOXA1 were shown in Fig. 6D, E. ATAC-seq suggested that the KCNK1 TSS region had an open chromatin structure and was in an active transcriptional state (Fig. 6B, C). Further, we collected histone modification ChIP-seq of labelled silencers (H3K27me3), promoters (H3K4me3) and enhancers (H3K4me1 and H3K27ac). The results suggested the existence of potentially active (promoters, enhancers) and repressive (silencers) genomic regulatory elements in the KCNK1 upstream TSS and the existence of a complex transcriptional regulatory network in KCNK1 (light red shaded areas in Fig. 6B, C). In addition, there were loops between the promoter of the KCNK1 TSS and the downstream super enhancer (SE) and the downstream typical enhancer (TE), suggesting that the promoter might be spatially engaged with the SE and TE to promote KCNK1 expression (light orange shaded region in Fig. 6B, C). GRHL2 and FOXA1 might also be spatially involved in the above transcriptional regulation (light blue shaded area in Fig. 6B, C). Among them, as a ChIP marker, RAD21 might help promoter-enhancer (P-E) interactions by regulating the spatial structure of chromatin to promote active transcription of genes.

### Potential pathway enrichment analysis

**Fig. 7:**
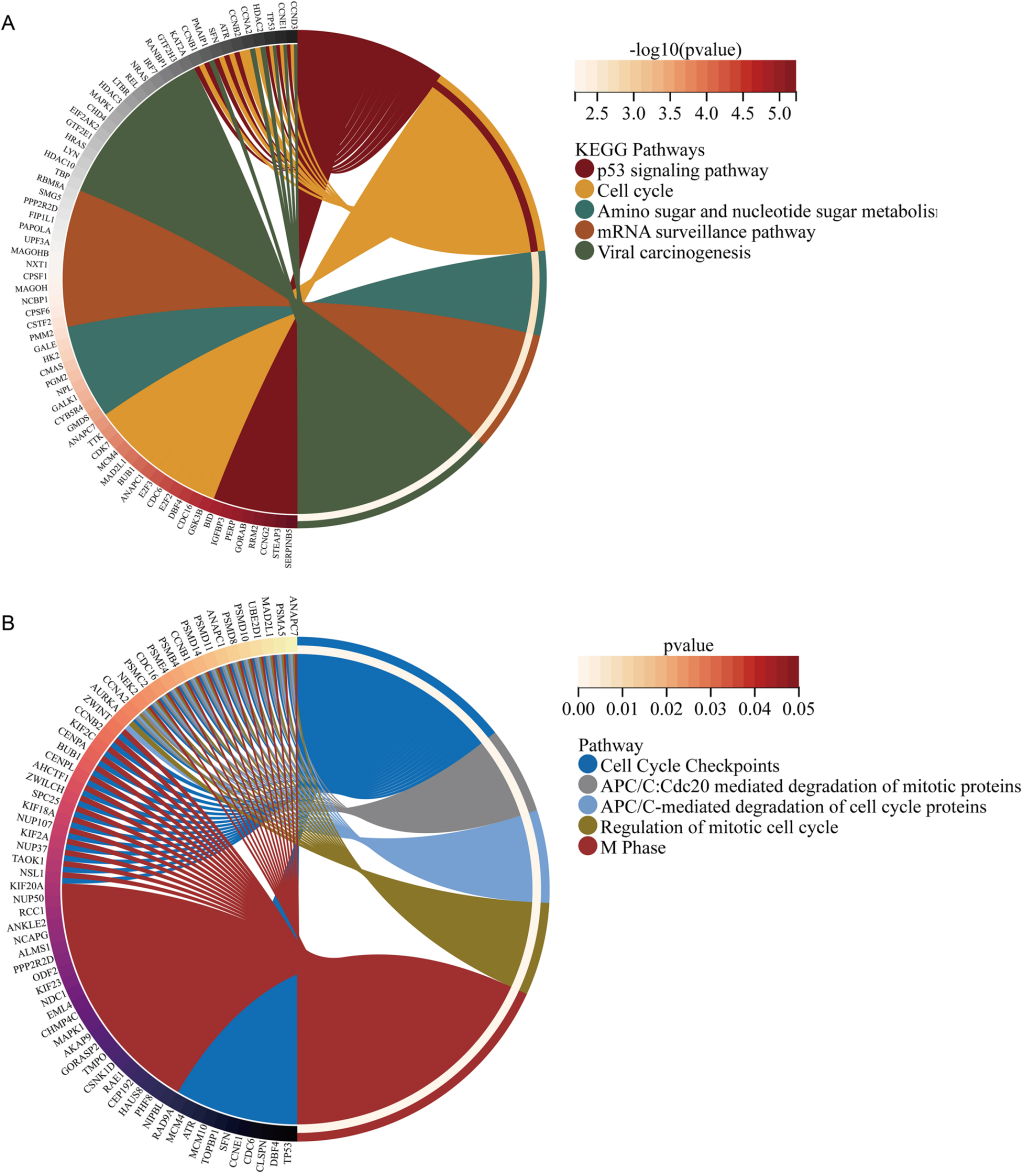
Pathway enrichment analysis of highly expressed co-expressed genes of KCNK1. **A** KEGG enrichment analysis. **B** Reactome enrichment analysis

In this study, there were 1154 HECEGs that could be screened for the next step of analysis. KEGG analysis showed that KCNK1-associated HECEGs were mainly enriched in signalling pathways such as p53 signaling pathway, Amino sugar and nucleotide sugar metabolism, mRNA surveillance pathway and Cell cycle (Fig. 7A). Reactome analysis suggested that HECEGs were mainly involved in APC/C-mediated degradation of cell cycle proteins, Cell Cycle Checkpoints, Regulation of mitotic cell cycle, M Phase and APC/C:Cdc20 mediated degradation of mitotic proteins were significantly enriched (Fig. 7B). GO analysis similarly showed that KCNK1-associated HECEGs were mainly involved in cell cycle-related signalling pathways (Additional file 2: Figure S4).

### Preliminary exploration of the molecular function of KCNK1 in BC

**Fig. 8:**
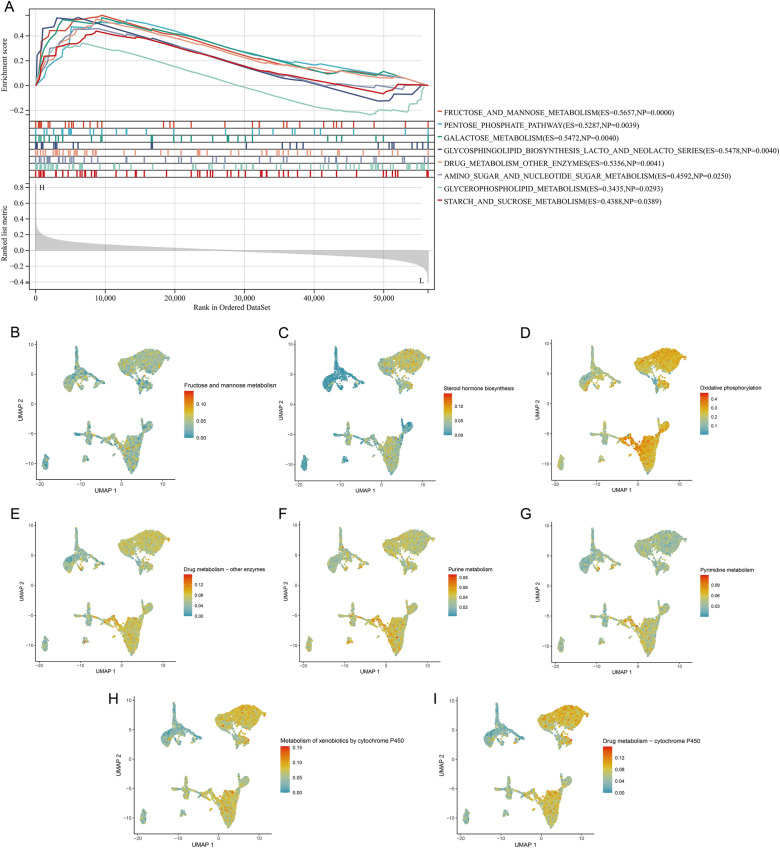
Preliminary validation of the molecular function of KCNK1. **A** Gene set enrichment analysis of KCNK1 in bladder cancer. **B**–**I** UMAP plots of single-cell metabolic activity scores

In addition, we tried to clarify the molecular mechanisms and biological pathways of KCNK1. Gene set enrichment analysis (GSEA) showed that KCNK1 was involved in multiple BC cell metabolism-related signalling pathways, especially glucose metabolism (Fig. 8A). Meanwhile, elevated KCNK1 expression could activate the activity of metabolic signalling pathways. Comparing with other cells, single-cell AUCell analysis showed that there were multiple active metabolic signalling pathways in BC epithelial cells, including fructose and mannose metabolism, Drug metabolism—other enzymes (Fig. 8B–I). The molecular mechanism of KCNK1 was further explored from protein interactions using PPI. As a hub gene of the interaction network, KCNK1 was involved in the formation of ion transmembrane transport proteins, especially potassium ions (Additional file 2: Figure S5).

### Correlation of KCNK1 expression with BC TME

**Fig. 9:**
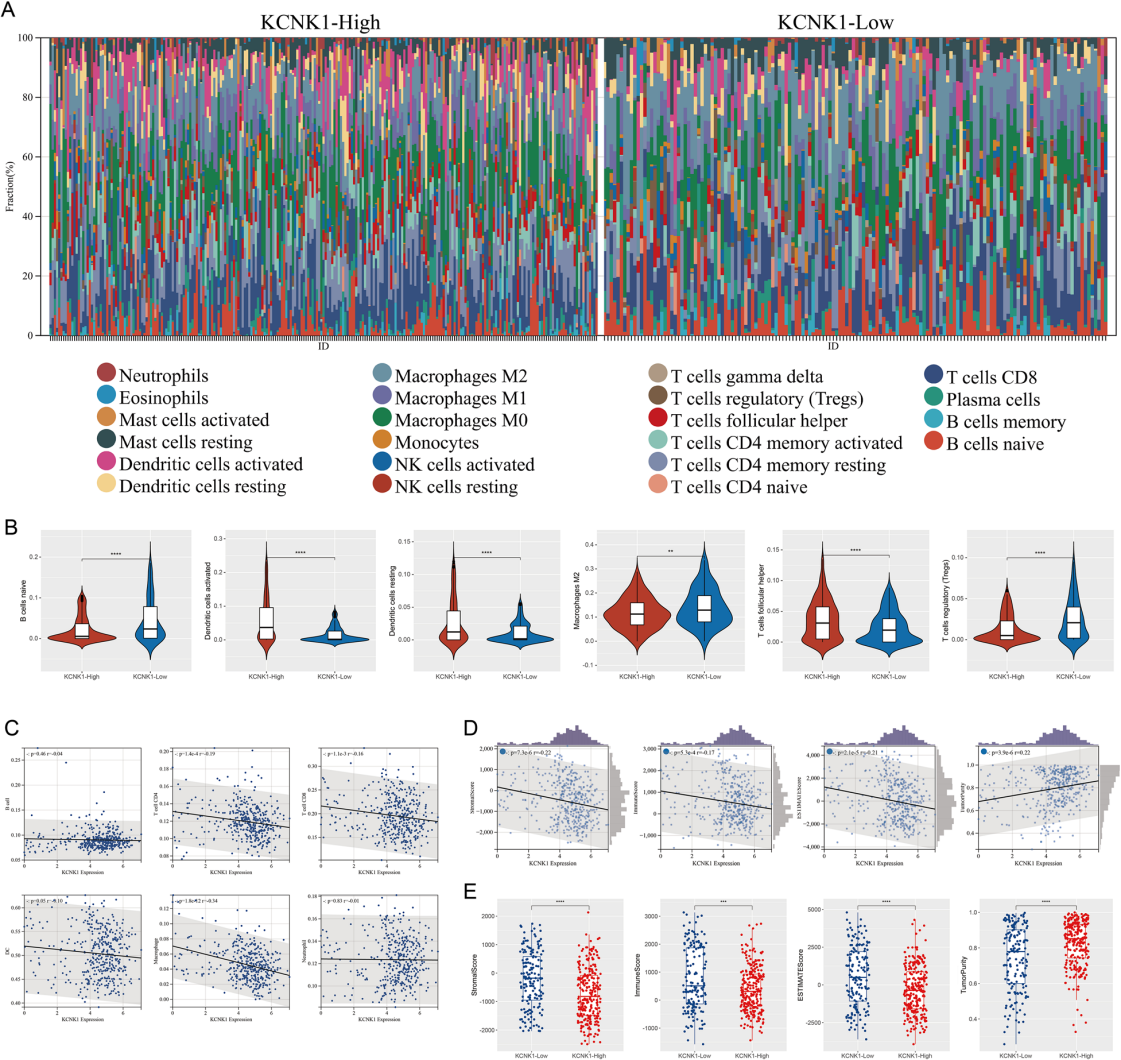
Significance of KCNK1 expression in the tumour microenvironment (TME). **A** TME composition of the high KCNK1 expression group and the low KCNK1 expression group. **B** KCNK1 expression was significantly correlated with the level of multiple immune cell infiltration. **C** Correlation of KCNK1 expression with multiple immune cells. **D** Correlation of KCNK1 expression with TME scores. **E** Significant differences in TME scores between the high KCNK1 expression group and the low KCNK1 expression group. ^ns/NS^p > 0.05, ^∗^p < 0.05, ^∗∗^p < 0.01, ^∗∗∗^p < 0.001, ^∗∗∗∗^p < 0.0001

TME has an essential role in immunosurveillance, immune escape, immunosuppression and immunotherapy [31]. Figure 9A demonstrated the composition of 22 immune-infiltrating cells in the high KCNK1 expression and low KCNK1 expression groups in BC TME. Comparing with the low KCNK1 expression group, the high KCNK1 expression group had higher levels of infiltration of Dendritic cells activated (*p* < 0.0001), Dendritic cells resting (*p* < 0.0001) and T cells follicular helper (*p* < 0.0001), and lower levels of infiltration of B cells naive (*p* < 0.0001), Macrophages M2 (*p* < 0.01) and T cells regulatory (Tregs) (p < 0.0001) (Fig. 9B). In addition to the two immune cells (B cell and Neutrophil), KCNK1 expression was negatively correlated with cellular levels of T cell CD4 (*r* = − 0.19; *p* = 1.4e-4), T cell CD8 (*r* = − 0.16; *p* = 1.1e-3), DC (*r* = − 0.10; *p* = 0.05), and Macrophage (*r* = − 0.34; *p* = 1.8e-12) (Fig. 9C). Further, TME was assessed as a whole, and we found that KCNK1 expression was significantly negatively correlated with TME stromal score (*r* = − 0.22; *p* = 7.3e-6), immune score (*r* = − 0.17; *p* = 5.3e-4) and ESTIMATE score (*r* = − 0.21; *p* = 2.1e-5), and positively correlated with tumour purity (*r* = 0.22; *p* = 3.9e-6) (Fig. 9D). TME scores were significantly different in the high and low KCNK1 expression groups (*p* < 0.05; Fig. 9E).

### Clinical value of KCNK1 expression in BC patients

**Fig. 10:**
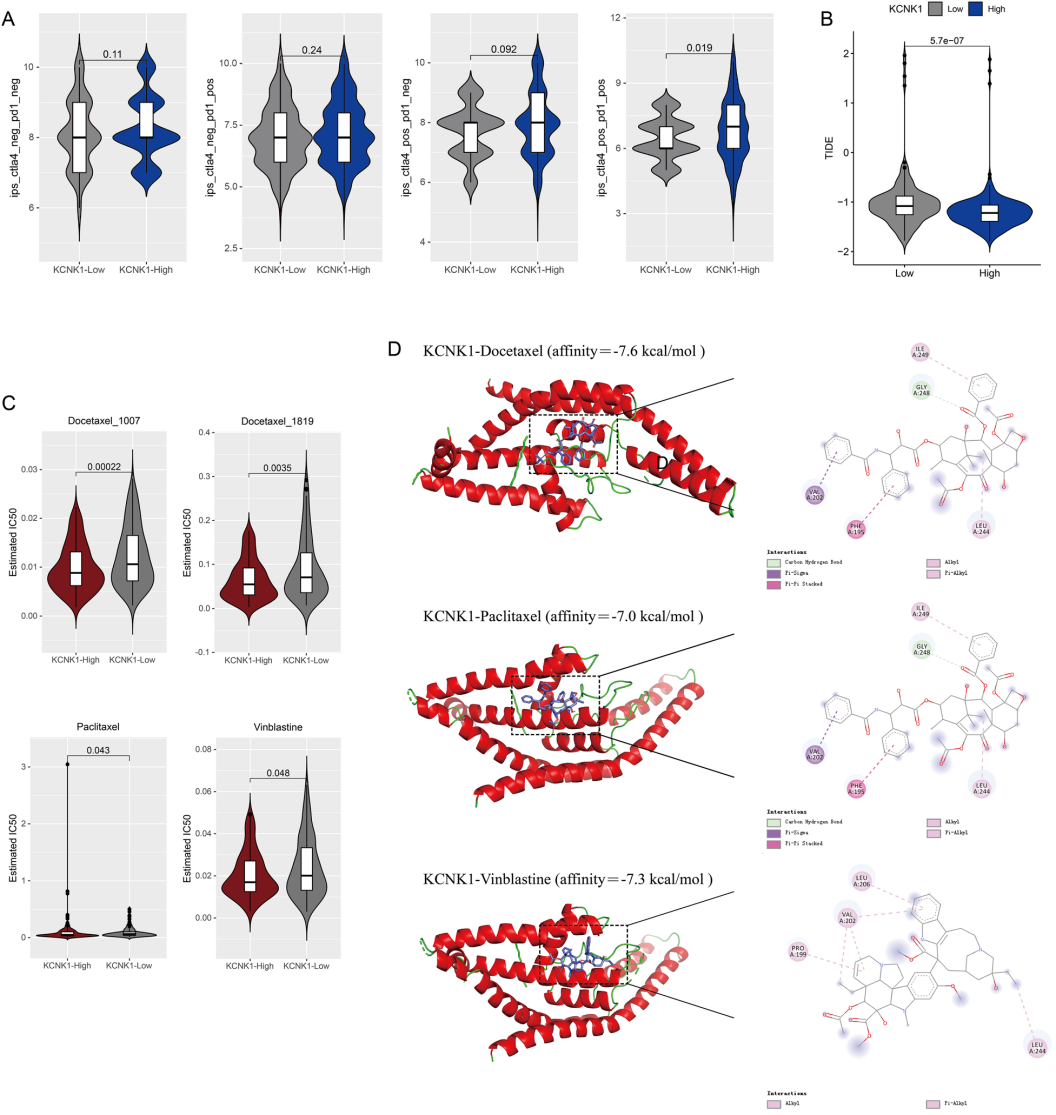
Clinical treatment and molecular docking of KCNK1 expression. **A** Immune-targeted therapy scores in the high KCNK1 expression group and low KCNK1 expression group. **B** Tumour immune escape scores in the high KCNK1 expression group and low KCNK1 expression group. **C** Assessment of the pharmacological therapeutic potential of KCNK1 expression. **D** Molecular docking to assess the affinity of KCNK1 protein to drugs

We also explored the clinical significance of KCNK1 overexpression in BC. Based on the clinicopathological characterisation information of the in-house samples, we found that KCNK1 expression was significantly associated with age, pathology grade and pathologic_M in BC patients (Table 2). Among them, KCNK1 expression in BC was significantly different in low-grade (Pathology Grade I) versus high-grade (Pathology Grade II and III&IV) (*p* < 0.05; Additional file 2: Figure S2B). Immune-targeted therapy suggested that patients in the high KCNK1 expression group had better outcomes when treated with the combination of anti-CTLA-4 and anti-PD-1 (*p* = 0.019; Fig. 10A). Meanwhile, the high KCNK1 expression group had lower TIDE scores, suggesting a low tumour immune escape potential and a better outcome of immunotherapy for BC patients (*p* = 5.7e-7; Fig. 10B). Further predicting potential therapeutic drugs for KCNK1, we found that the drug half maximal inhibitory concentration (IC50) of docetaxel, paclitaxel, and vinblastine was low in the high KCNK1 expression group (*p* < 0.05; Fig. 10C). Molecular docking showed that KCNK1 protein had a high molecular affinity for docetaxel (affinity = − 7.6 kcal/mol), paclitaxel (affinity = − 7.0 kcal/mol) and vinblastine (affinity = − 7.3 kcal/mol) and possessed better drug efficacy (Fig. 10D).

**Table 2:** Study of the relationship between KCNK1 mRNA expression and clinical characteristics of KCNK1 patients from in-house data

Clinical features	KCNK1 mRNA Expression			*t* (t-test) or *F*(ANOVA test)	*p* value
	Number	Mean	SD		
Age				− 2.246	0.026
> 60	114	9.7930	2.44224		
≤ 60	85	8.9459	2.86712		
Sex				− 1.365	0.181
Male	174	9.5149	2.71142		
Female	25	8.8480	2.21606		
Pathology grade				4.099	0.018
Grade I	21	8.0381	2.95812		
Grade II	92	9.5435	2.61026		
Grade III and Grade IV	76	9.8158	2.30426		
Pathologic_M				− 13.763	< 0.0001
M0	195	9.3785	2.65985		
M1	2	12.0000	0.0E0		
Pathologic_N				0.256	0.775
N0	186	9.4495	2.64476		
N1	9	8.8000	2.56125		
N2	3	9.3333	4.61880		
Pathologic_T				2.374	0.072
T1	53	8.9208	2.50610		
T2	101	9.5208	2.63394		
T3	36	10.1667	2.36039		
T4	8	8.0500	4.27852

## Discussion

This was a comprehensive study involving KCNK1 expression, molecular mechanism, biological function and clinical value in BC. A large number of samples (n = 1486) were included in this study identifying significantly elevated KCNK1 mRNA expression in BC and 245 in-house samples were stained by IHC to validate this result at the protein level. Secondly, chromatinomics techniques were used to analyse potential regulatory mechanisms of KCNK1 expression. Based on scRNA-seq analysis, the expression and cellular communication of KCNK1 in BC were further determined in different cells. Immediately thereafter, we explored the molecular functions and biological pathways of KCNK1. Furthermore, we discovered that KCNK1 overexpression in BC was significantly associated with TIME and was helpful in predicting drug treatment efficacy and clinicopathological features.

Aberrantly expressed KCNK1 was significantly associated with poor prognosis in a variety of cancers, such as BRCA [20], thyroid cancer [16], lung [32] and pancreatic adenocarcinomas [19]. However, aberrantly expressed KCNK1 in BC has never been reported. In the present study, we identified that KCNK1 mRNA expression was significantly elevated in BC using extensive gene microarray and sequencing data from a global multicentre database, which was validated by in-house IHC experiments. This reflects that this study follows the concept of comprehensive evaluation of evidence in evidence-based medicine. Chromatin genomics revealed the presence of transcriptionally active regions and active/repressed gene regulatory elements in the KCNK1 TSS, and that GRHL2 and FOXA1 bound to the KCNK1 TSS. There were loops between promoters on KCNK1 TSS and downstream SE and TE. Taken together, we suggested that RAD21 might lead to P-E interactions in the KCNK1 TSS in three-dimensional space by regulating the spatial structure of chromatin. At the same time, KCNK1 TSS was transcriptionally regulated by GRHL2 and FOXA1, which activated the corresponding gene regulatory elements and finally promoted the elevated KCNK1 expression in BC.

Abnormal cell metabolism and cell cycle progression could lead to cancer development [33, 34]. In this study, we found that HECEGs of KCNK1 were mainly involved in the cell cycle pathway, and KCNK1 was closely related to the transmembrane transport of potassium ions. Previous studies have reported that, as an important gene encoding a potassium channel subunit protein, KCNK1 could control ion channels to participate in a variety of cellular activities [35, 36]. Ion channels could promote the deterioration of cancer cells and then accelerate malignant cell proliferation [16]. Therefore, we suggested that KCNK1 could promote the abnormal proliferation of cancer cells by activating potassium ion channel activity. Further, we found that BC epithelial cells abnormally accumulated large amounts of KCNK1 and had multiple active metabolic signalling pathways. Notably, GSEA suggested that the high expression of KCNK1 in BC could activate the activity of multiple metabolic signalling pathways, which corresponded to the results of single-cell analysis. Excitingly, cellular communication suggested that BC epithelial cells were involved in the cell cycle by engaging in the MK signalling pathway (MDK-dominated L-R pair), which was thought to underlie the involvement in the cell cycle being required [37]. Metabolic pathways could support key events in different phases of the cell cycle [38]. It was reported that MK could activate downstream signalling pathways (e.g. PI3K/Akt, MAPK and JAK/STAT), which played important roles in cell proliferation and differentiation [39–41]. Therefore, KCNK1 might mediate the abnormal cell cycle and metabolism of BC epithelial cells by controlling membrane ion channels, which ultimately promoted the proliferation of cancer cells.

TME has received widespread attention in promoting tumour progression and drug resistance [42, 43]. Ion channels had a key role in regulating the acid–base balance and metabolism of the TME, thereby affecting the activity and function of immune cells [44, 45]. Among them, potassium channels could inhibit the anti-tumor ability of T cells and tumor-associated macrophages by increasing potassium ion concentration [46–48]. However, there were no reports on KCNK1 in BC TME as an important coding gene for potassium channels. In the present study, we found that high KCNK1 expression in BC was closely associated with low infiltration levels of T cell CD4, T cell CD8, DC and Macrophage. Meanwhile, elevated KCNK1 expression resulted in lower stromal scores, immune scores, and ESTIMATE scores and higher tumour purity in TME. Therefore, we speculated that high KCNK1 expression might increase BC TME potassium ions, thereby reducing the level of multiple immune cell infiltration and increasing tumour purity.

Considering the significance of KCNK1 in BC immunoassays, we further analysed the correlation between KCNK1 expression and immunotherapy. In this study, the high KCNK1 expression group had lower TIDE scores and significantly higher in the two-drug combination treatment. It is well known that PD-1/PD-L1 and CTLA-4 inhibitors are common immune checkpoint inhibitors [49]. Ong et al. found that high potassium ions upregulated the expression of the immune checkpoint protein PD-1 in T cells [50]. Therefore, KCNK1 might reduce the potential for tumour immune escape and enhance immune response (anti-CTLA-4 and anti-PD-1 combination therapy) by modulating elevated potassium ions. Secondly, we found that BC patients had lower IC50 and greater drug molecule affinity for docetaxel, paclitaxel and vinblastine in the high KCNK1 expression group. The three drugs mentioned above were reported to work by blocking the progression of the cell cycle, which ultimately led to the death of the cancer cells [51, 52]. Paclitaxel and docetaxel might affect the proliferation of BRCA cells by modulating potassium currents, and TME could induce docetaxel resistance [42, 53, 54]. We concluded that KCNK1 could affect the cell cycle via potassium channels, thereby increasing drug sensitivity of BC patients to docetaxel, paclitaxel, and vincristine.

Certain limitations remain inevitable. Specifically, in vivo and in vitro experiments are needed to further explore the potential molecular biological functions and clinical value of KCNK1 in BC. In addition, we need more in-house high-throughput techniques to validate the potential epigenetic regulatory mechanisms of KCNK1. Nevertheless, the present study revealed that KCNK1 was highly expressed in BC at both mRNA and protein levels, as well as the transcriptional regulatory mechanism of KCNK1. More importantly, we proposed the view that KCNK1 affected the BC cell cycle, cellular metabolism, and TME by regulating potassium channels. This helps to increase our understanding of KCNK1 in BC and provides new insights into the occurrence and development of BC.

## Conclusion

This study integrated in-house samples and multicentre data to identify that KCNK1 was highly expressed in BC and predominantly distributed in BC epithelial cells. A complex and sophisticated three-dimensional spatial transcriptional regulatory network existed in the KCNK1 TSS and promoted elevated KCNK1 expression. The high expression of KCNK1 might be involved in the cell cycle, cellular metabolism and TME through the regulation of potassium channels, which ultimately contributed to the deterioration of BC.

### Supplementary Information

**Additional file 1: **Raw data from internal immunohistochemical staining.

**Additional file 2: Figure S1.** Flow of bladder cancer related dataset screening in this study. **Figure S2.** The ability of KCNK1 to distinguish BC samples and control samples, and the correlation between KCNK1 expression and pathology grade. **A** Receiver operator characteristic curve. **B** Box plot. **Figure S3.** Afferent/efferent global communication patterns of multiple bladder cancer cell clusters. **Figure S4.** GO enrichment analysis of highly expressed co-expressed genes of KCNK1. **Figure S5.** KCNK1-based protein-protein interaction networks as well as functional pathways.

## Data Availability

All data relevant to this study are provided in the article or in Additional file 1, and do not need to be deposited in public databases.
