# Correction: Overexpressed KCNK1 regulates potassium channels affecting molecular mechanisms and biological pathways in bladder cancer

**DOI:** 10.1186/s40001-024-01905-5

**Published:** 2024-06-20

**Authors:** Wei Zhang, Xiao‑Song Chen, Ying Wei, Xiao‑Min Wang, Xian‑Jin Chen, Bang‑Teng Chi, Lin‑Qing Huang, Rong‑Quan He, Zhi‑Guang Huang, Qi Li, Gang Chen, Juan He, Mei Wu

**Affiliations:** 1 Department of Pathology, The First Affiliated Hospital of Guangxi Medical University, 6 Shuangyong RD, Nanning, 530021 Guangxi Zhuang Autonomous Region People’s Republic of China; 2 Department of Medical Oncology, The First Affiliated Hospital of Guangxi Medical University, 6 Shuangyong RD, Nanning, 530021 Guangxi Zhuang Autonomous Region People’s Republic of China

**Correction: European Journal of Medical Research (2024) 29:257** 10.1186/s40001-024-01844-1

In this article [1] Wei Zhang^1†^, Xiao-Song Chen^1†^, should have been denoted as an equally contributing authors.

The original article has been corrected.
