# Supplementary material for: Overexpressed KCNK1 regulates potassium channels affecting molecular mechanisms and biological pathways in bladder cancer
Source: Eur J Med Res. 2024 Apr 30;29:257. doi: 10.1186/s40001-024-01844-1 (PMC11059691; doi:10.1186/s40001-024-01844-1)
Supplement: Supplementary file 2 — Additional file 2: Figure S1. Flow of bladder cancer related dataset screening in this study. Figure S2. The ability of KCNK1 to distinguish BC samples and control samples, and the correlation between KCNK1 expression and pathology grade. A Receiver operator characteristic curve. B Box plot. Figure S3. Afferent/efferent global communication patterns of multiple bladder cancer cell clusters. Figure S4. GO enrichment analysis of highly expressed co-expressed genes of KCNK1. Figure S5. KCNK1-based protein-protein interaction networks as well as functional pathways. [file 40001_2024_1844_MOESM2_ESM.docx]

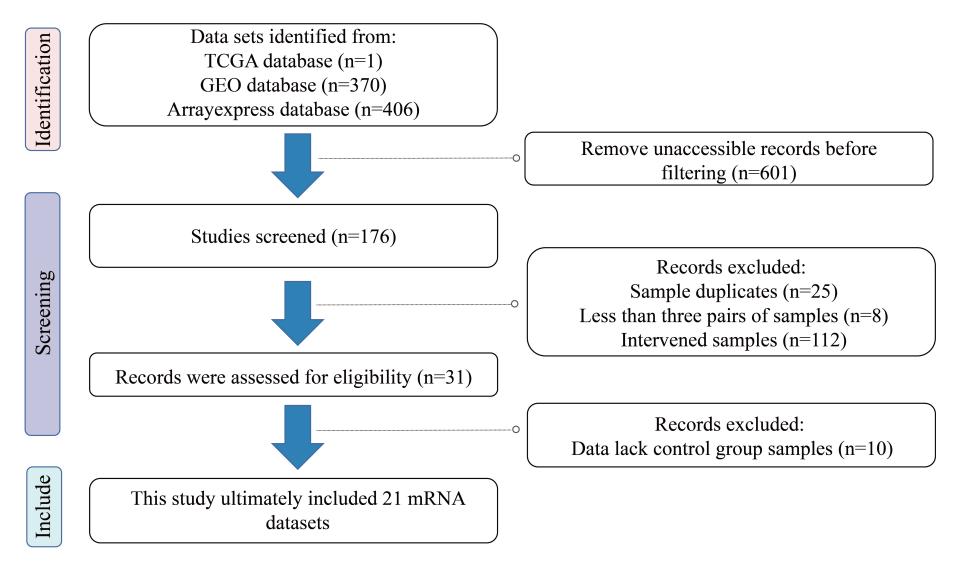


**Figure S1** Flow of bladder cancer related dataset screening in this study.


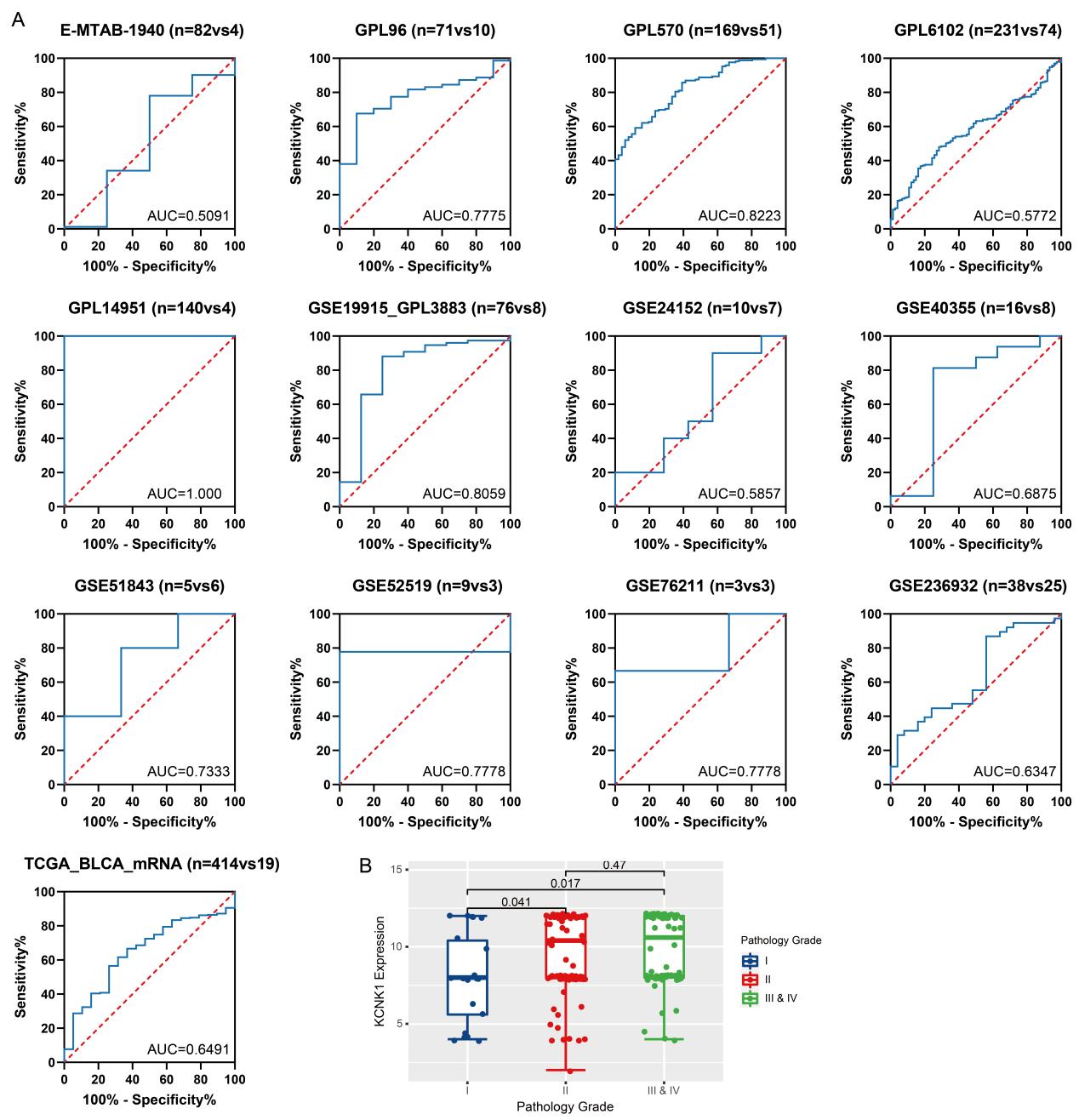


**Figure S2** The ability of KCNK1 to distinguish BC samples and control samples, and the correlation between KCNK1 expression and pathology grade. (A) Receiver operator characteristic curve. (B) Box plot.


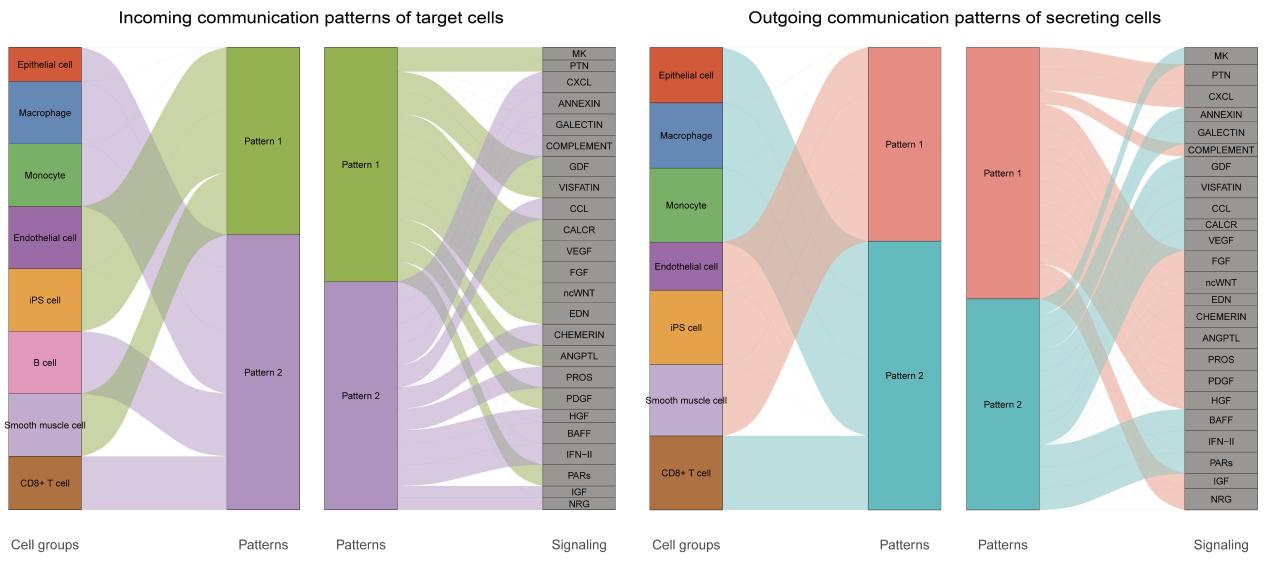


**Figure S3** Afferent/efferent global communication patterns of multiple bladder cancer cell clusters.


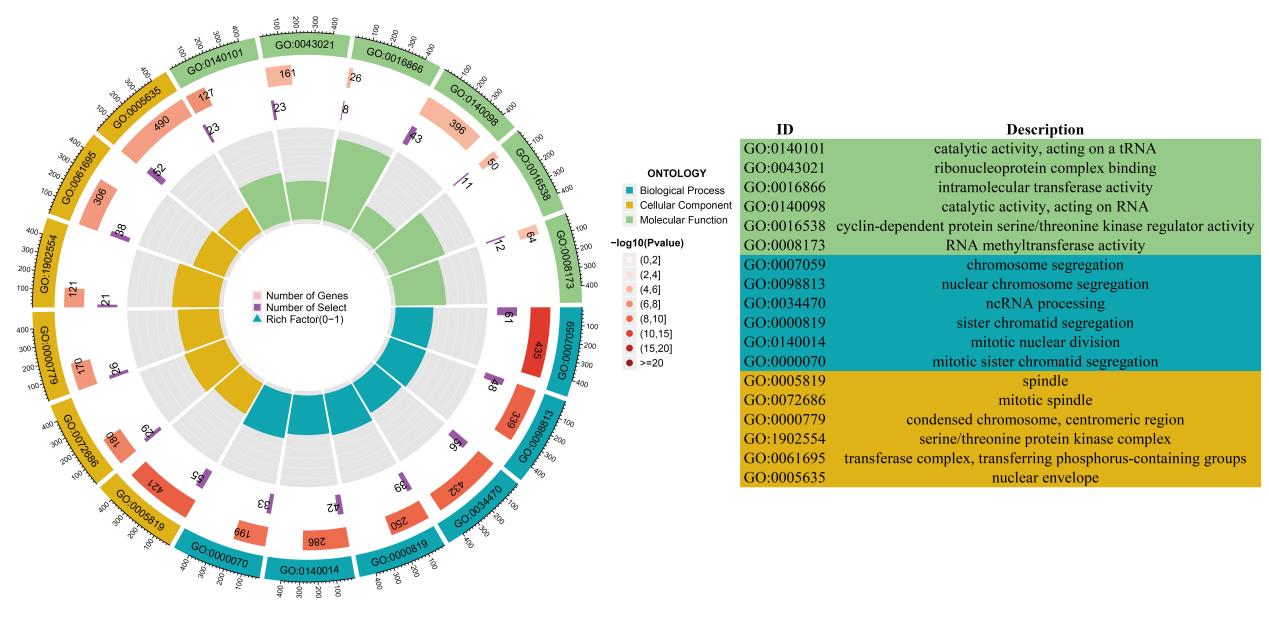


**Figure S4** GO enrichment analysis of highly expressed co-expressed genes of KCNK1.


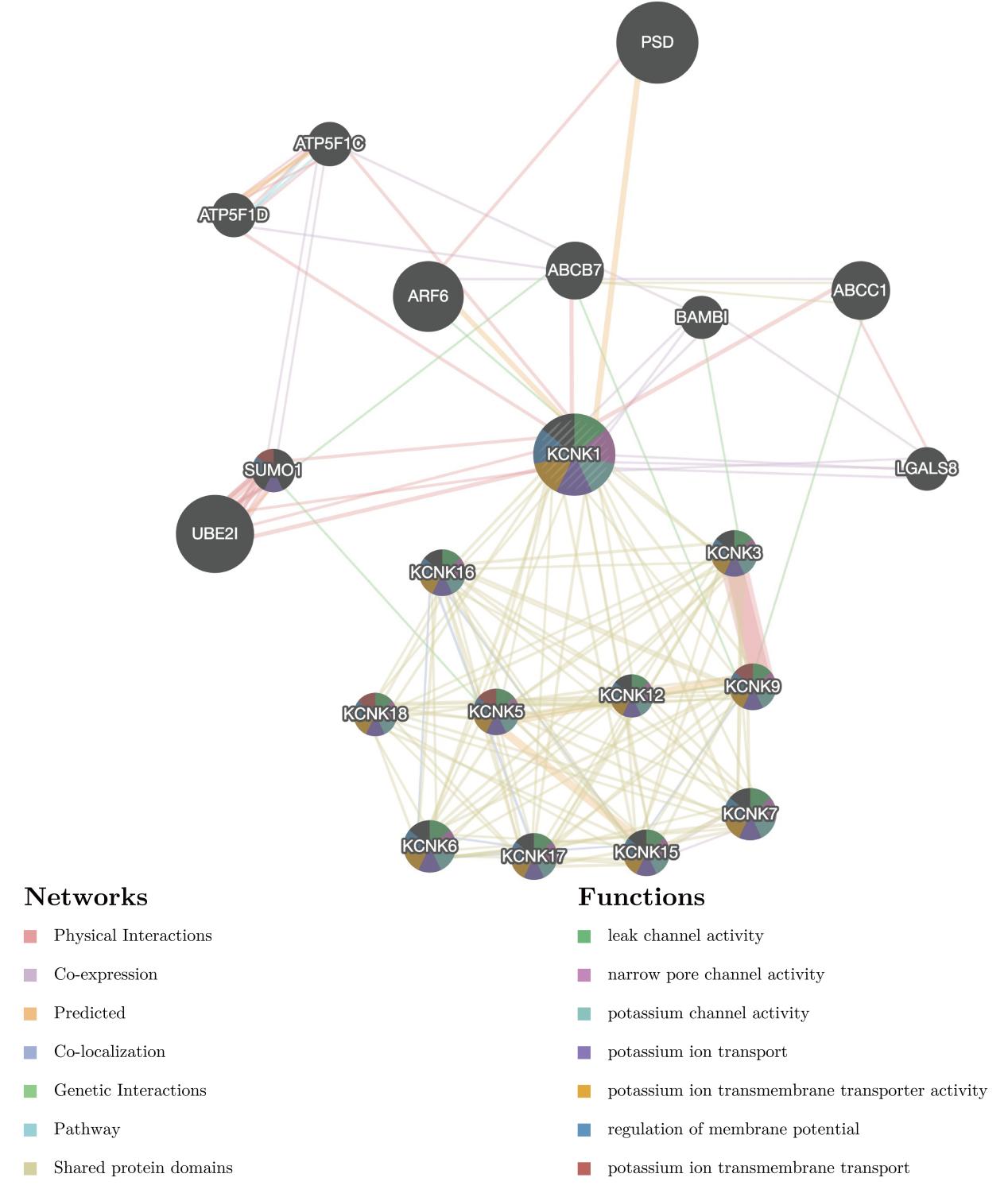


**Figure S5** KCNK1-based protein-protein interaction networks as well as functional pathways.
